# Evaluating the Experiences of Personal Safety of Foundation Year Doctors on Psychiatry Placement in South London and Maudsley NHS Foundation Trust

**DOI:** 10.1192/bjo.2026.11272

**Published:** 2026-06-30

**Authors:** Frances Weston, Rakeb Yoseph, Arabella Borgstein, Mosopefoluwa Ajegbomogun, Leila Frodsham

**Affiliations:** Guy’s and St Thomas’ Hospital, London, United Kingdom

## Abstract

**Aims:**

In 2023, 75% of resident psychiatry doctors (n=210) in Southeast England had experienced verbal abuse, 50% physically threatened, and only 33% felt confident in workplace security measures. A similar survey was carried out within South London and Maudsley NHS Foundation Trust (SLAM) in 2023, which found 70% of Foundation Doctors had experienced verbal or physical abuse from patients. Measures were put in place to improve safety, however were not clearly defined, and a review of their effectiveness has not yet occurred. This research aimed to bridge this gap by understanding the safety measures currently in place, and to determine whether doctors feel safe within their psychiatry placements.

**Methods:**

We conducted a cross-sectional survey of SLAM Foundation Doctors on psychiatry placements between August 2025 to February 2026, to explore perceptions of safety during their training. The survey was distributed electronically and comprised of both quantitative and qualitative items, including closed-ended and free-text questions. Using this data, we identified areas that need improvement, and now aim to implement strategies to aid in improving foundation doctor experiences.

**Results:**

A total of 8 doctors responded to the survey. 62.5% of doctors feel unsafe or somewhat safe in their psychiatry placement. 87.5% did not receive any personal safety or breakaway training on starting their job. Only 25% of doctors received a safety induction for their ward or hospital, including how to locate emergency buzzers and staff alarms. 62.5% of doctors have been verbally assaulted, and report experiencing something they found traumatic during the rotation.

Qualitative data highlighted fears surrounding being left alone with acutely unwell patients due to a lack of safety induction and information on personal safety alarms.

**Conclusion:**

Our research highlights that a high proportion of foundation doctors report feeling unsafe during their psychiatry placements. Most respondents did not receive personal safety or breakaway training, and only a minority received a local safety induction. Qualitative findings reinforced these findings, highlighting concerns around being left alone with acutely unwell patients, alongside a lack of safety and de-escalation training. Experiencing violence or aggression alongside a lack of safety training may adversely affect doctors’ experiences, engagement with learning opportunities, and ability to deliver a high standard of care. Identifying gaps in safety provision is therefore essential to inform targeted quality improvement initiatives and enhance both doctor training experiences and patient outcomes.

